# Surgery for rare adenosquamous carcinoma of the scrotum: A case report and literature review

**DOI:** 10.1097/MD.0000000000032994

**Published:** 2023-02-22

**Authors:** Yexiao Sun, Xihong Miao, Lei Wang, Yanfeng Zhang, Zhihua Shen

**Affiliations:** a Department of Dermatology, Chengde Central Hospital, Hebei, China; b Department of Urology, Chengde Central Hospital, Hebei, China.

**Keywords:** adenosquamous carcinoma, chemotherapy and radiotherapy, scrotum, case report

## Abstract

**Rationale:**

Adenosquamous carcinoma of the scrotum is a rare cancer associated with poor prognosis. It is diagnosed through the presence of both adenocarcinoma and squamous cell carcinoma.

**Patient concerns:**

It may be difficult to diagnose at early stages and may have poor survival.

**Diagnoses:**

We report a case of adenosquamous carcinoma of the scrotum in a 58-year-old male patient who presented with left scrotal mass for >1 year.

**Interventions:**

This is the first case in the literature of primary adenosquamous carcinoma of the scrotum managed successfully with surgery and post-surgery chemotherapy and radiotherapy.

**Outcomes:**

The patient remained disease-free for 10 months postoperatively.

**Lessons:**

The surgery treatment combined with postoperative radiotherapy and chemotherapy can improve the survival of adenosquamous carcinoma.

## 1. Introduction

Adenosquamous carcinoma is a subtype of squamous cell carcinoma with adenocarcinoma differentiation. Scrotal squamous cell carcinoma is relatively rare in clinical practice, with an age-standardized incidence between 0.34 and 0.44 per 10,000,000 male person-years and an overall 5-year relative survival of 77%.^[1,2]^ The subtype of adenosquamous carcinoma is even rare, its incidence is unclear due to a historical lack of diagnostic criteria. According to the World Health Organization, adenosquamous carcinoma contains at least 10% of each of the malignant squamous and glandular components, and the 2 components differentiate in the same tissue in different regions.^[3]^ Usually, squamous cell carcinomas are predominant, and adenocarcinoma components can be tubular or glandular. Immunohistochemistry can contribute to the diagnosis of adenosquamous carcinoma. Adenosquamous carcinoma is usually highly malignant and aggressive, and 80% of patients have lymph node metastasis or systemic metastasis at the time of diagnosis. In a literature review of 93 cases, regional metastases occurred in 47.4% of cases and distant metastases in 24.7%.^[4]^ Here, we report a case of scrotal adenosquamous carcinoma of unknown etiology and discuss its diagnosis and treatment based on the literature.

## 2. Case presentation

**Figure 1.**
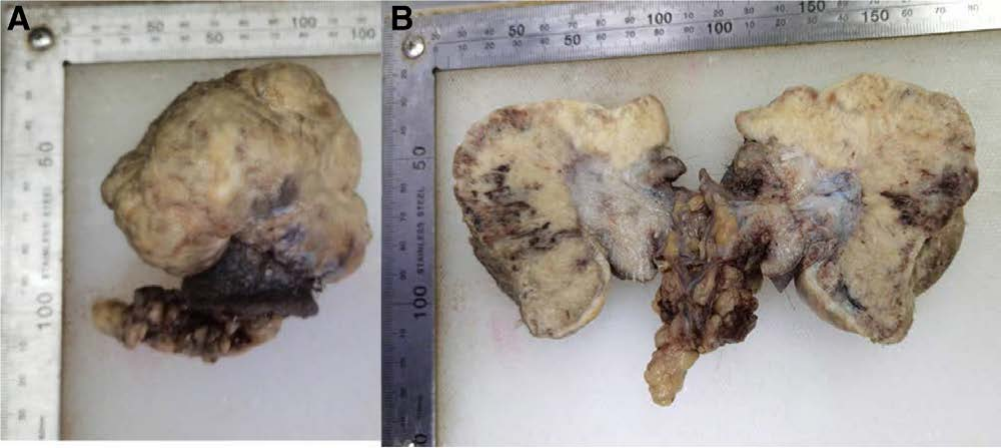
The macroscopic examination of the tumor. (A) Overall tumor appearance after excision: the tumor was about 10*10*11 cm in size, a pale pink cauliflower-like mass, with a pale color locally and an unsmooth surface. (B) Appearance of the cut surface: the cut surface is gray-white and gray-yellow.

**Figure 2.**
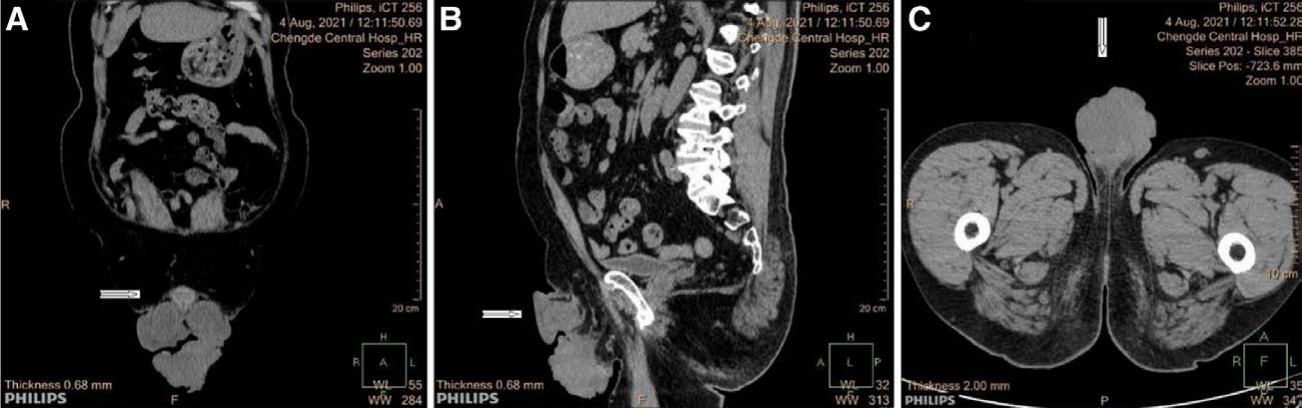
CT of the tumor. (A) In the coronal plane, (B) sagittal plane, and (C) horizontal plane, large tumor tissue and lymph node metastasis can be seen. CT = computed tomography.

**Figure 3.**
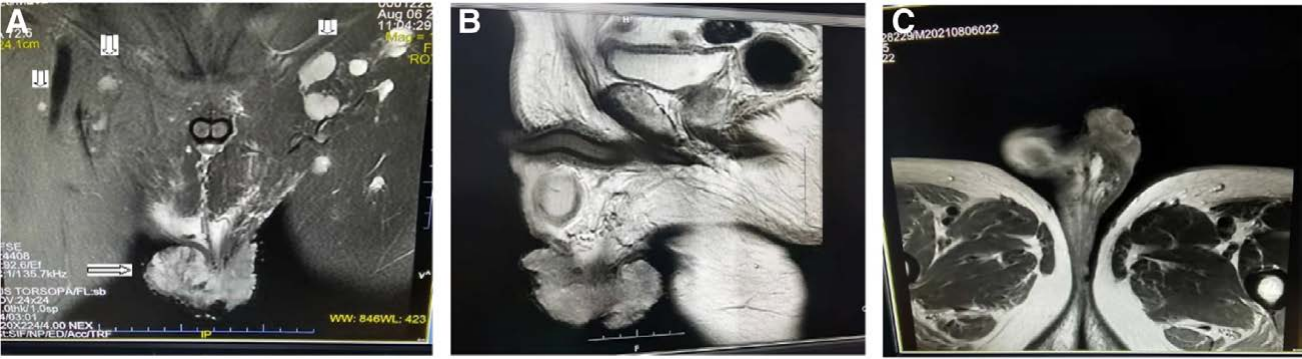
MRI of the tumor. (A) Coronal plane, (B) sagittal plane, and (C) horizontal plane showed a malignant scrotal mass with bilateral inguinal lymph node metastasis. MRI = magnetic resonance imaging.

**Figure 4.**
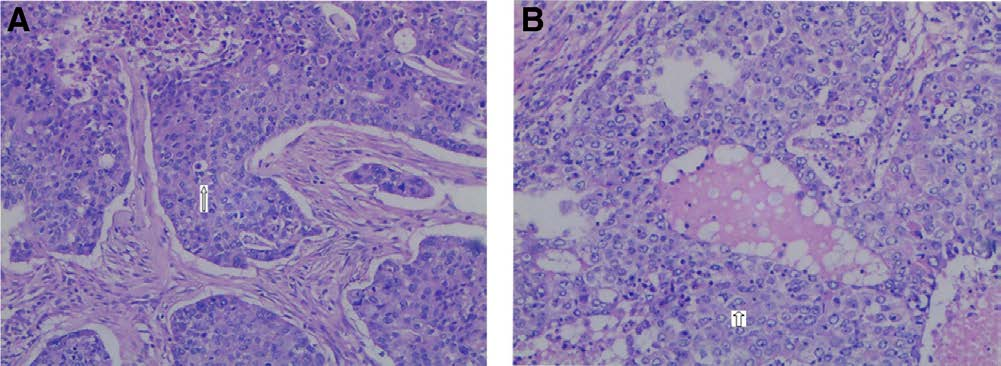
H&E staining (*100). (A) Shows the differentiation of squamous cell carcinoma; the tumor shows invasive growth, the cell nests are distributed in sheets, and the cells are moderate-to-severe dysplasia. (B) Shows adenocarcinoma differentiation; the tumor grows infiltrating, cystic, and lumen-like, with moderate-to-severe cellular dysplasia with visible nucleoli.

**Figure 5.**
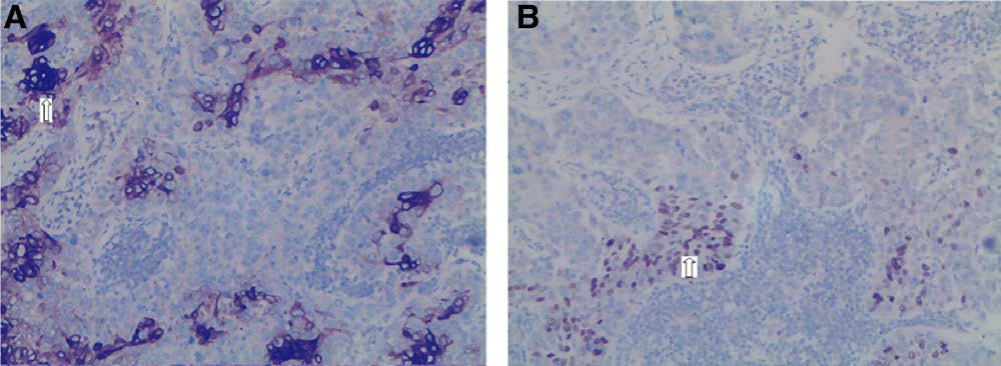
Immunohistochemistry (*100) (A) Immunohistochemistry for CK7, arrows indicate adenocarcinoma differentiation. (B) Immunohistochemistry for P40, arrows indicate squamous cell carcinoma differentiation. CK7 = creatine kinase 7.

A 58-year-old male patient initially presented to our tertiary hospital with a 1-year history of a mass in the left scrotum in August 2021. He reported having the exogenous mass for >1 year without treatment, and the mass was neither painful nor itchy, while gradually increasing in size, causing difficulty in walking. Physical examination showed a light pink cauliflower-like mass in the middle of the left scrotum, near the raphe of the scrotum, about 10 × 10 × 11 cm in size; the surface was not smooth, with redness, swelling and ulceration, and no obvious tenderness (Fig. 1). The patient had a type 2 diabetes history; there was no similar medical history in the family. Laboratory tests including routine tests of blood, urine, and feces showed no obvious abnormality. Computed tomography scan of the kidney and ureter showed a malignant mass in the scrotum, bilateral scrotal effusion, multiple enlarged lymph nodes in the groin and multiple retroperitoneal lymph nodes on both sides, and prostate calcification (Fig. 2). Further magnetic resonance imaging of the penis revealed a malignant scrotal mass with bilateral inguinal lymph node metastases (Fig. 3). The pathological examination of the skin lesions showed that the cancer cells had multifocal invasion under the epidermis of the left scrotum, forming a nest-like or cord-like distribution, with large, deep-stained nuclei, strange shapes, and pathological mitoses. Necrosis and keratinization were occasionally seen in the squamous cell carcinoma part, and the glandular differentiated part had cyst-like or glandular-like structures with vacuolated nuclei and mucus secretion (Fig. 4). No cancer cells were found at the base of the skin and the incision margin. Immunohistochemical results showed creatine kinase (CK) (+), CK5/6 (partial+), P40 (partial+), CK7 (partial+), carcinoembryonic antigen (partial−), GATA binding protein 3 (−), S-100 (−), Vimentin (−), CK20 (−), and cluster of differentiation 56 (−), and Ki67 proliferation index was 80% (Fig. 5). The patient was then diagnosed with adenosquamous carcinoma of the scrotum. He received tumor resection in a referral hospital on August 9, 2021, and robot-assisted laparoscopic bilateral inguinal lymph node dissection and bilateral pelvic lymph node dissection on October 9, 2021. Adverse events of left lower extremity edema and scrotal effusion occurred postoperatively, and the effusion puncture and catheter drainage were performed. The patient received 4 cycles of chemotherapy (paclitaxel (nab bound) 0.4 g ivgttdl, carboplatin 500 mg ivgttdl/q21d) from December 2021 to February 2022, and he reported no pain at the end of chemotherapy. On March 2022, the patient had a Karnofsky score of 70 and started to receive radiotherapy, with a dose of 50.4Gy/1.8Gy/28F. Until we submit the case report, the wounds have healed, and the patient remained disease free and was still under close follow-up.

## 3. Discussion

Scrotal squamous cell carcinoma is very rare, and its etiology has been confirmed to be multifactorial, including but not limited to long-term exposure to substances containing chemical carcinogens such as arsenic, chronic inflammation, human papillomavirus infection, etc.^[4,5]^ In the case reported here, the patient was a bus driver, and the scrotum environment was humid and stuffy due to prolonged sitting, which was a chronic irritant.

Scrotal adenosquamous carcinoma is an extremely rarely differentiated subtype of squamous cell carcinoma. The lack of clinically relevant and validated staging criteria for adenosquamous carcinoma increases the difficulty of diagnosis. It was previously reported that the actual diagnostic accuracy of clinical cervical adenosquamous carcinoma was 57.6%.^[6]^ In appearance, scrotal adenosquamous carcinoma typically presents as a smooth-surfaced scrotal nodule,^[7]^ but may also present as an ulcerative and invasive tumor that is indistinguishable from squamous or basal cell carcinoma by the naked eye. Adenocarcinoma differentiation is seen in the deep layers and is focal or widespread within the tumor. The specific staining of adenosquamous carcinoma has dual features of squamous cell carcinoma (pan-keratin-positive, epithelial membrane antigen-positive) and adenocarcinoma (carcinoembryonic antigen-positive). Microscopically, squamous differentiation often originates in the epidermis, forming keratinizing cysts or keratin beads.^[8,9]^ Glandular differentiation is in well-differentiated squamous nests, glands are lined by cuboid or low columnar epithelium, and ductal differentiation is sometimes highlighted by eosinophilic stratum corneum.^[10,11]^ In our case, the morphology of the tumor and immunohistochemical results support the diagnosis of scrotal adenosquamous carcinoma.

Scrotal squamous cell carcinoma is highly malignant and aggressive, with local invasion or early lymph node metastasis, and poor prognosis. Early diagnosis of cancer, and no local tissue invasion and metastasis, may have a better prognosis. According to whether the tumor tissue invades or metastasizes, Ray and Whitmor propose scrotal carcinoma staging: Stage A: A1 – disease localized to the scrotum; A2 – locally extensive disease involving adjacent structures (penis, perineum, testis, and/or cord structures, pubic bone) by continuity but without evident metastasis. Stage B: regional metastases, resectable; Stage C: regional metastases, non-resectable; Stage D: distant metastasis (beyond regional nodes).^[12]^ In this case, abdominal ultrasonography, lower abdominal and chest computed tomography plain scan, lower abdominal magnetic resonance imaging, and other examinations found that although the lesion did not involve surrounding tissues or had distant metastasis, bilateral inguinal lymph node metastasis occurred, it was considered a Phase D case.

The treatment for squamous cell carcinoma of the scrotum is surgical resection of the diseased tissue with a margin of at least 2 to 3 cm of normal tissue. The primary tumor should be excised from the surrounding subcutaneous tissue. If the cancer does not invade, it is not necessary to remove the scrotal contents. If the invasion exceeds the reticular layer, unilateral resection of the scrotal contents is required. Regarding the management of inguinal lymph nodes, the efficacy of regional lymph node dissection is still controversial, and bilateral radical inguinal dissection is generally recommended to remove micrometastases.^[13]^ In our case, due to bilateral lymph node metastases from the right scrotal tumor, surgical lymph node dissection was difficult. Therefore, we adopted palliative treatment, that is, wide excision of the scrotal tumor tissue, preservation of the testis, and dissection of the left inguinal lymph node. Thereafter, robotic-assisted laparoscopic bilateral inguinal lymph node dissection and bilateral pelvic lymph node dissection were performed.

The prognosis of scrotal squamous cell carcinoma is related to whether the tumor can be completely removed at the initial operation and whether there is lymph node metastasis.^[14]^ If inguinal lymph nodes are involved, the 5-year survival rate is approximately 25%. In this case, the patient had a relatively long course of the disease and underwent surgical resection 1 year after the tumor was discovered. During surgery, inguinal lymph node metastases were found. In order to maximize the removal of tumor cells, robot-assisted endoscopic bilateral inguinal lymph node dissection and bilateral pelvic lymph node dissection were performed. Complete excision of the skin lesions was limited due to the extensive range of skin lesions and their occurrence in the scrotum. Therefore, surgery combined with chemotherapy and radiotherapy is used to remove tumor lesions and prevent recurrence. Four cycles of chemotherapy with paclitaxel and carboplatin were performed in the same year after the operation, followed by a course of radiotherapy. At present, the scar on the patient’s surgical site has healed, and there is no indication of distant metastasis. The patient is recommended for lifelong follow-up.

## 4. Conclusion

The surgery treatment combined with postoperative radiotherapy and chemotherapy can improve the survival of adenosquamous carcinoma.

## Acknowledgments

We are grateful for the technical support from the Department of Urology and Pathology, Chengde Central Hospital

## Author contributions

**Conceptualization:** Zhihua Shen.

Data curation: Xihong Miao.

Visualization: Lei Wang.

Writing – original draft: Yexiao Sun.

Writing – review & editing: Yanfeng Zhang, Zhihua Shen.
